# Modeling the Genetic Control of HIV-1 Dynamics After Highly Active Antiretroviral Therapy

**DOI:** 10.2174/138920208784340777

**Published:** 2008-05

**Authors:** Chang-Xing Ma, Yao Li, Rongling Wu

**Affiliations:** 1 Department of Biostatistics, University of Buffalo, Buffalo, NY 14214, USA; 2 Department of Statistics, University of Florida, Gainesville, FL 32611, USA

**Keywords:** Hardy-weinberg equilibrium, bi-exponential function, quantitative trait loci, HIV dynamics, functional mapping.

## Abstract

The progression of HIV disease has been markedly slowed by the use of highly active antiretroviral therapy (HAART). However, substantial genetic variation was observed to occur among different people in the decay rate of viral loads caused by HAART. The characterization of specific genes involved in HIV dynamics is central to design personalized drugs for the prevention of this disease, but usually cannot be addressed by experimental methods alone rather than require the help of mathematical and statistical methods. A novel statistical model has been recently developed to detect genetic variants that are responsible for the shape of HAART-induced viral decay curves. This model was employed to an HIV/AIDS trial, which led to the identification of a major genetic determinant that triggers an effect on HIV dynamics. This detected major genetic determinant also affects several clinically important parameters, such as half-lives of infected cells and HIV eradication times.

## 1. INTRODUCTION

A current hope for avoiding the fatal consequences of HIV-1 infection lies in treatments with highly active antiretroviral therapy (HAART), which consists of combinations of three or more drugs that inhibit HIV reverse transcriptase or protease [1]. When patients are treated with HAART, the load of virions (plasma HIV-1 RNA copies) detectable in their blood plasma usually drops sharply. Although considerable variation has been observed in the dynamics of the change of viral load among patients [2-4], little is known about the control mechanisms of genes in regulating viral decay with time.

The change of viral loads with time is a dynamic process, whose genetic control may also follow a temporal pattern. The genetic mapping of a dynamic trait has proven to be very difficult due to its high-dimensionality, but a key issue related to dynamic mapping has been resolved at the University of Florida by proposing a statistical model, called functional mapping [5]. Functional mapping integrates the mathematical equations of a biological process into a general genetic mapping framework in which the tests for genetic control are based on curve parameters that specify the dynamic pattern. In this article, we extend the idea of functional mapping to detect a major genetic determinant predisposing to the decline of viral load after initiation of HAART by incorporating the mathematical analysis of HIV dynamics. We further show how the genetic determinant detected is activated to affect HIV dynamics and how it controls clinically important variables in AIDS/HIV trials. 

## 2. METHODS

In general, the amount of virions after initiation of treatment with potent antiretroviral agents follows a certain dynamic pattern that can be characterized by mathematical functions. Based on a firm understanding of biological interactions using real virological data from clinical trials, some simplified forms of HIV dynamic models have been derived [6]. Fig. (**1**) illustrates the dynamics of viral load for 53 HIV-1-infected patients after the treatment with HAART from the AIDS Clinical Trials Group (ACTG) Protocol 315, supported by the National Institute of Allergy and Infectious Diseases. Viral loads of these patients were measured on days 0, 2, 7, 10, 14, 21, 28 and weeks 8 and 12. This ACTG 315 dataset is of typical longitudinal nature and has been subjected to extensive statistical analyses [6, 7]. In particular, Wu and Ding [6] have shown that HIV-1 dynamics for the ACTG 315 data can be adequately fitted by a biexponential function, expressed as

(1) $$V\left(t\right)=\lambda_{1}{\text{e}}^{{-d}_{1}t}+\lambda_{2}{\text{e}}^{{-d}_{2}t}$$

where and λ_1_ and λ_2_ represent the initial viral production rates in two different biological compartments, productively infected cells and long-lived and/or latently infected cells, and  *d*_1_ and *d*_2_ are the decay rates of these two compartments, respectively. Different combinations of the curve parameters (λ_1_, λ_2_, *d*_1_, *d*_2_) determine the shapes of the dynamic change of HIV-1 after the treatment with HAART. 

Whether or not there exists a major genetic determinant to control HIV-1dynamics or curves in hosts can be tested by using a *functional mapping *model proposed to map a longitudinal trait [5, 8]. The hypothesis tests embedded within the functional mapping context allow for the exploration of the control mechanism of agenetic determinant in regulating the time-dependent curve changes of HIV-1 load. The statistical foundation of functional mapping is based on the mixture model in which each curve fitted by a series of measurements at *τ*  time points for any subject, arrayed by **y  =**  [*y*(1),…, *y*(*τ*)], is assumed to have arisen from one of a known or unknown number of components, each component being modeled by a multivariate normal distribution density. Assuming that there are *L *genotypes contributing to the variation among different curves, this mixture model is expressed as

(2) $$\text{y}∼p\left(\text{y}\left|\pi ,\varphi ,\eta \right.\right)=\pi_{1}f\left(\text{y};\varphi_{1},\eta \right)+\cdot \cdot \cdot +\pi_{L}f\left(\text{y};\varphi_{L},\eta \right),$$

where *π* = (*π*_1_,…, *π_L_*)^T^ are the mixture proportions (i.e., genotype frequencies) which are constrained to be non-negative and sum to unity; *φ* = (*φ*_1_,…, *φ_L_*)^T ^are the component-(or genotype) specific parameters, with *φ*_1_ being specific to component *l*; and *η*  is parameters which are common to all components. The likelihood function of longitudinal data measured for a random sample of size *n *is constructed as

(3) $$L\left(\pi ,\varphi ,\eta \left|\text{y}\right.\right)=\prod_{i=1}^{n}\left[\pi_{1}f\left({\text{y}}_{i};\varphi_{1},\eta \right)+\cdot \cdot \cdot +\pi_{L}f\left({\text{y}}_{i};\varphi_{L},\eta \right)\right],$$

where all samples are assumed to have independent responses to times. Traditional mapping methods [9] can be readily extended to accommodate the multivariate nature of time-dependent traits. The multivariate normal distribution of individual *i* measured at *τ *time points is expressed as

(4) $$f\left({\text{y}}_{i};\varphi_{l},\eta \right)=\frac{1}{{\left(2\pi \right)}^{\tau /2}{\left|\Sigma \right|}^{1/2}}\exp\left[-\frac{1}{2}\left({\text{y}}_{i}-{\text{m}}_{l}\right)\Sigma^{-1}{\left({\text{y}}_{i}-{\text{m}}_{l}\right)}^{\text{T}}\right],$$

where y*_i_* = [*y_i_* (1),…, *y_i_* (*τ*)] is a vector of observation measured at *τ* time points and **m**_*l*_ = [*μ_l_* (1),…,* μ_l _*(τ)] is a vector of expected values for genotype *l* at different points. At a particular time *t*, the relationship between the observation and expected mean can be described by a linear regression model,

$y_{i}(t)=\sum_{l=1}^{L}x_{\mathrm{il}}\mu_{l}(t)+e_{i}(t)$

where *x_il_* is the indicator variable denoted as 1 if a genotype *l *is considered for subject * i *and 0 otherwise; *e_i _*(*t*) is the residual error that is iid normal with the mean of zero and the variance of *σ*^2^ (*t*). The errors at two different time points, *t*_1_ and *t*_2_, are correlated with the covariance of cov(*t*_1_*, t*_2_). These (co)variances comprise a (*τ*  × *τ*) matrix Σ.

The use of a traditional multivariate mapping approach to map genes for a longitudinal trait is limited in three aspects: (1) individual expected means of different genotypes at all points and all elements in the matrix Σ need to be estimated, resulting in substantial computational difficulties when the vector and matrix dimension is large; (2) the result from this approach may not be biologically meaningful because the underlying biological principle for the longitudinal trait is not incorporated; and (3) this approach cannot be well deployed on a practical scheme because of (2). Thus, some biologically interesting questions cannot be asked and answered. 

The functional mapping model includes two tasks: (1) model the time-dependent expected means of genotype *l* using a mathematical function, such as Equation 1 for HIV-1dynamics; and (2) model the structure of the within-subject (co)variance matrix using the autoregressive [AR(1)] model, expressed as 

$\sigma^{2}(1)=\cdot \cdot \cdot =\sigma^{2}(\tau )=\sigma^{2}$

for the variance, and 

$\mathrm{cov}(t_{1},t_{2})=\sigma^{2}\rho^{\left|t_{1}-t_{2}\right|}$

for the covariance between any two time intervals *t*_1_ and *t*_2_, where 0 < *ρ* < 1 is the proportion parameter with which the correlation decays with time lag. The incorporation of these tasks into the likelihood function leads to the significant reduction of the parameter number. With this incorporation, we will estimate the curve parameters and matrix parameters arrayed by (*λ*_1_, *λ*_2_,*d*_1_,*d*_2_, *ρ*, *σ*^2^) rather than individual genotypic means at different time points for HIV-1 progression mapping.  

We also need to derive a procedure for estimating the genotype frequencies (*π*) for the HIV-1 dynamics genetic determinant in a sample of patients drawn from a natural population. For a mapping project, these genotype frequencies can be expressed as the conditional probabilities of the genotypes of interest conditional upon the known marker genotypes. But for the ACTG 315 data in which marker genotypes have not been collected yet, we can only test whether there is a latent segregating major gene for HIV-1 dynamics. Suppose there is a major genetic determinant with two alleles *A *and *a *that affects HIV-1 dynamics. Let *q *and 1 - *q *denote the allele frequencies for *A *and *a*, respectively. The three genotypes at the major gene, *AA, Aa *and *aa*, have genotypic frequencies expressed as *π*_1 _= *q*^2^ + *D*, *π*_2 _= 2*q*(1-*q*) – 2*D*, and *π*_3 _= (1 – *q*)^2^ + *D*, respectively, where *D *is the coefficient of Hardy-Weinberg disequilibrium at the major gene. For an arbitrarily chosen patient, he or she must carry one (and only one) of the three genotypes, with a probability represented by *π*_1_, *π*_2_ or *π*_3_. The mixture model for all individuals based on these three latent genotypes establishes a theoretical foundation for characterizing an individual’s major genotype. More recently, we have derived a closed-form solution for the EM algorithm to estimate the genotypic frequencies of a major genetic determinant in a population [10].  

## 3. RESULTS AND DISCUSSION

**Fig. (1):**
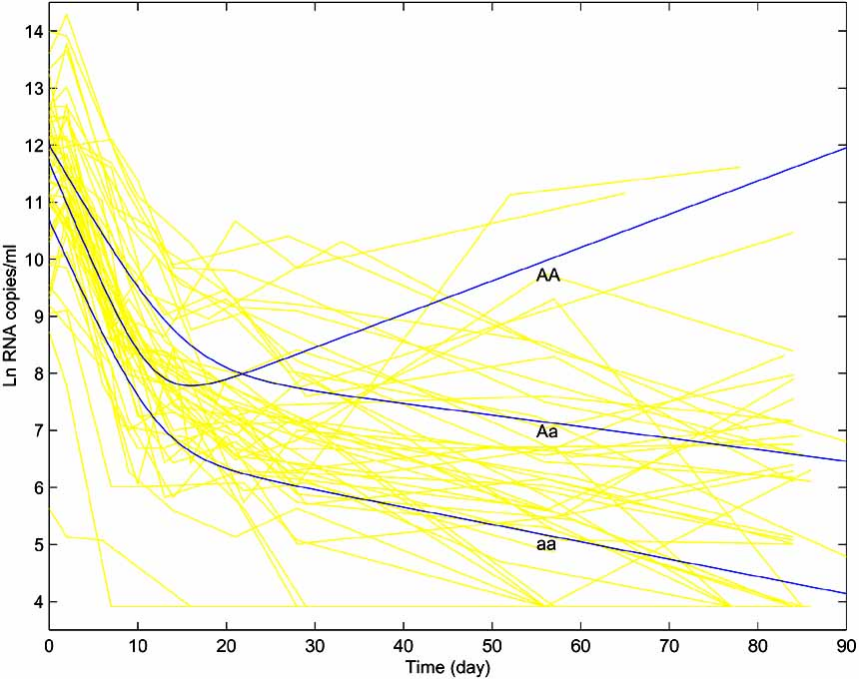
Load trajectories of HIV-1 virions (measured in viral RNA copies) for 53 patients (with shadow curves) during the first 12 weeks after the initiation of HAART for the ACTG 315 data. HIV dynamics can be adequately fitted by a biexponential equation. The three thick curves each represent a different genotype (denoted by *AA, Aa* or *aa*) at a major genetic determinant detected by the functional mapping approach and can better explain the data than a single population mean curve (*LR* = 31.42 > critical threshold of 24.67 at  *α* = 0.01 estimated from 1000 simulation replicates). The curves of the three genotypes are described by *V*_2_(*t*) = *e*^11.6959-0.3677*t*^ + *e*^6.6988+0.0584*t*^ for genotype *AA*, *V*_1_(*t*) = *e*^11.9814-0.2726*t*^ + *e*^8.2763+0.0202*t*^ for genotype *Aa*, and *V*_0_(*t*) = *e*^10.6641-0.3477*t*^ + *e*^6.8662-0.0303*t*^ for genotype *aa*. Note that the two homozygotes can be arbitrarily assigned by *AA* or *aa* in this example because no marker information is provided. The hypothesis tests for the genotypic differences of *d*_1_ and *d*_2_ suggest that the major gene detected affects the turnovers of HIV in two different virus compartments.

By incorporating the bi-exponential function of HIV-1 dynamics into the EM-implemented mapping framework, we have successfully detected a putative host major genetic determinant responsible for HIV dynamics in the ACTG data. The log-likelihood ratio (*LR*) test statistic (31.42) for the full model (there is a major gene) over the reduced model (there is no major gene) was calculated, and it is much greater than the critical threshold (24.67, *P*=0*.*0001) determined from simulated data under the null hypothesis that there is no major gene. However, it is possible that the three classifications of viral load response curves identified from our model may also be due to other treatment factors, like ages and sex. A mixed model incorporating these factors is proposed from which the evidence for the existence of a major genetic determinant is observed. The three genotypes at the detected major genetic determinant displayed marked differentiations in their viral load trajectories (Fig. **1**). The heterozygote (*Aa*) and one homozygote (denoted by *aa*) that together account for an overwhelming majority of patients (~90%) were found to decline consistently with time in viral load after initiation of antiretroviral drugs. For the second homozygote (*AA*) in the frequency of ~10%, viral load turns out to increase from day 20 following a short period of decline after the drug administration. We estimated allele frequencies for the major genetic determinant detected, which are 0.32 for the allele causing the increase of viral load after day 20 and 0.68 for the allele maintaining a consistent decline. No significant Hardy-Weinberg disequilibrium (*D*) was detected according to the log-likelihood ratio test. 

The two decay rates of different virus compartments, *d*_1_ and *d*_2_, have clinical values in AIDS trials [7]. The half-lives of productively infected cells (*t*_1/2_ = log 2/*d*_1_) were estimated as (1.9, 2.5, 2.0) days for three major genotypes *AA, Aa *and *aa*, respectively. The corresponding *t*_1/2_ values of long-lived and/or latently infected cells (log 2/*d*_2_) were estimated as (11.9, 34.4, 22.9) days. While the major genetic determinant detected has a nonsignificant effect on the half-life of productively infected cells (*LRd*_1 _=3*.*29, d.f. = 2, *P* > 0.05), it triggers a significant effect on the half-life of long-lived and/or latently infected cells (*LRd_2_*= 10*.*30, d.f. = 2, *P *< 0*.*01). The control of the detected major genetic determinant over the half-times of long-lived and/or latently infected cells suggests a possibility of altering second-phase viral load trajectories through a gene therapy strategy.  

We calculated the eradication times for the two compartments, productively infected cells and long-lived and/or latently infected cells, approximated by *τ*_1 _= log *λ*_1_ / *d*_1_ and *τ*_2 _= log *λ*_2_ / *d*_2_, respectively [7]. The estimated eradication times for the three QTL genotypes are (6.7, 9.1, 6.8) days for the first virus compartment and (32.6, 104.8, 63.6) days for the second virus compartment. We found that the major genetic determinant detected displayed significant effects on the eradication times for the two compartments (*LRτ*_1 _=6*.*84, d.f. = 2, *P* < 0.05 and *LRτ*_2 _= 13*.*19, d.f. = 2, *P* < 0.01). The genetic regulation of eradication times may assist clinicians in optimizing drug therapy on the basis of each patient’s genetic constitution.

Due to the staggering number of people infected with HIV (40 million), it is important to develop the most potent drugs to quickly eliminate all HIV from the blood and from the body. The detection of a major genetic determinant for HIV dynamics strongly suggests that HIV infection can be more efficiently prevented and curbed only after personalized strategies of gene therapy have been designed based on the gene a particular patient carries. The genetic determination of the life spans of infected cells during different phases implies that the virus may be fully eradicated through the development of individualized therapy.
